# Bile Duct Replacement in Hepatobiliary Surgery: *A Systematic Review*

**DOI:** 10.1002/wjs.70078

**Published:** 2025-09-24

**Authors:** Mehdi Boubaddi, Chetana Lim, Eric Savier, Claire Goumard, Florence Jeune, Geraldine Rousseau, Filomena Conti, Fabiano Perdigao, Olivier Scatton, the Liver Institute of la Pitié‐Salpêtrière (LIPS)

**Affiliations:** ^1^ Assistance Publique Hôpitaux de Paris Sorbonne Université Service de Chirurgie Hépato‐biliaire et Transplantation Hépatique Hôpital Pitié‐Salpêtrière Paris France; ^2^ Unité de Recherche Clinique SSPC Université de Picardie‐Jules Verne Amiens France; ^3^ Centre de Recherche de Saint‐Antoine (CRSA) INSERM UMRS‐938 Paris France; ^4^ Assistance Publique Hôpitaux de Paris Sorbonne Université Service d’Hépatologie Hôpital Pitié‐Salpêtrière Paris France

**Keywords:** biliary, hepatic, liver, transplant

## Abstract

**Background:**

Roux‐en‐Y hepaticojejunostomy (RYHJ) is currently the standard surgical technique for reestablishing biliary continuity, but it exposes the patient to serious biliary complications, such as anastomosis stricture and ascending cholangitis.

**Methods:**

The literature on biliary replacement using vein grafts as autologous substitutes according to the different stages of the IDEAL framework was reviewed.

**Results:**

Innovative biliary replacement techniques using bile duct substitutes have yet to reach stage 2a (development) of the IDEAL framework. Vein grafts are the most frequently used substitutes in animals and human studies. Twenty‐three patients have undergone bile duct reconstruction using four different substitutes: vein grafts (*n* = 13), omentum/round ligament (*n* = 5), jejunum (*n* = 4), and Teflon (*n* = 1). Biliary replacement using an autologous vein graft (*n* = 13) was performed for bile duct injury following cholecystectomy (*n* = 12) or hepatectomy (*n* = 1). The 90‐day mortality rate was zero. Morbidity occurred within 90 days in two patients (15.4%). Twelve patients were alive at the last follow‐up.

**Conclusions:**

Autologous venous graft as a substitute for biliary replacement may be an appealing alternative to RYHJ in hepatobiliary surgery, but this technique is still being developed.

## Graphical abstract

Roux‐en‐Y hepaticojejunostomy (RYHJ) is currently the standard surgical technique for reestablishing biliary continuity, but it exposes the patient to serious biliary complications, such as anastomosis stricture and ascending cholangitis. RYHJ also limits access to the biliary tree for endoscopic intervention in cases with biliary complications, making the percutaneous approach, with its inherent morbidity and impaired quality of life, the sole route for access

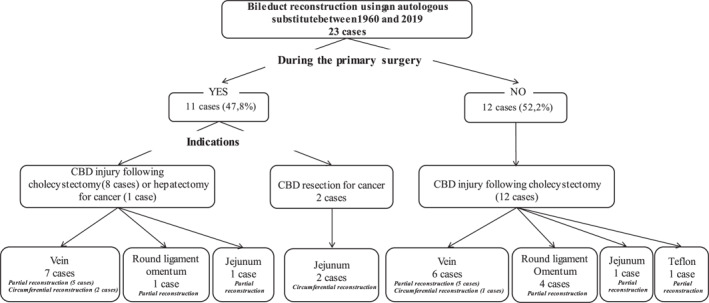

## 1 Introduction

Roux‐en‐Y hepaticojejunostomy (RYHJ) or Roux‐en‐Y choledochojejunostomy has been the treatment of choice for reestablishing biliary continuity since Robert Dahl reported the first RYHJ in 1909 [1]. The indications for RYHJ include benign diseases (injury, choledochal cysts, stricture, or obstruction), malignant tumors (perihilar cholangiocarcinoma), and liver transplantation in some situations.

RYHJ involves the nonphysiological reestablishment of biliary continuity, which results in loss of the sphincter of Oddi and can expose patients to biliary complications, such as ascending cholangitis (2.5%–23%) and strictures (10%–20%) [2, 3, 4, 5, 6, 7, 8, 9]. The long‐term effects of repeated cholangitis associated with chronic inflammation lead to the development of secondary biliary cirrhosis in 2.5%–11% of cases [10, 11]. RYHJ also limits access to the biliary tree for endoscopic intervention in cases with biliary complications, making the percutaneous approach, with its inherent morbidity and impaired quality of life, the sole route for access [12]. Refinements in RYHJ include the use of antireflux valves in the Roux‐en‐Y limb to reduce the risk of ascending cholangitis [13] and hepaticojejunostomy using short limb Roux‐en‐Y reconstruction to allow endoscopic access to the biliary tree [14].

The IDEAL guidelines provide a basis for reporting and evaluating new surgical innovations, moving through five stages (Idea, Development, Exploration, Assessment, and Long‐term studies) [15, 16]. Common bile duct replacement is an innovative surgical technique serving as a substitute for physiological reconstruction of the biliary tract. This technique originally used part of the posterior rectus sheath fascia with the peritoneum as an autologous substitute to surround the internal biliary drain, with the first case reported in 1916 [17]. In this context, some innovative surgeons reported the use of a vein graft as an easily available autologous substitute for reconstructing the common bile duct, with the first case reported in 1964 [18]. This paper reviews the literature and evaluates whether the use of a vein graft as an autologous substitute for bile duct reconstruction in hepatobiliary surgery has progressed in line with the IDEAL recommendations.

## 2 Methods

This study is a systematic review of the English language literature, including preclinical and human studies, in the setting of biliary reconstruction using vein grafts. This systematic review was conducted on November 2, 2024 and followed the Preferred Reporting Items for Systematic Reviews and Meta‐analyses (PRISMA) guidelines.

### 2.1 Objectives

The goals of this review are to provide a brief historical overview of bile duct reconstruction using vein grafts, to describe its feasibility and safety, and to assess whether bile duct reconstruction using vein grafts has progressed in line with the IDEAL framework.

### 2.2 Literature Search Strategy

A literature search was performed on the online databases, including PUBMED, MEDLINE, and EMBASE, by 2 of the authors (M.B and C.L). A specific search strategy was performed using the following keywords and/or MESH terms: “bile duct replacement,” “bile duct substitute,” and “bile duct reconstruction”. No time restriction was applied. Only experimental studies in animals and human clinical cases describing a technique for replacing the main bile duct were eligible for inclusion.

### 2.3 Study Selection and Data Extraction

Two authors (M.B and C.L) independently screened the titles and abstracts of the retrieved studies for relevance. All disagreements were resolved by discussion with a third author (O.S). The authors performed a full‐text review of the selected articles. In addition, the reference lists from the included studies were crosschecked to identify additional studies. For each patient, the following data were collected: etiology of initial biliary disease, location of the biliary reconstruction (common bile duct or intrahepatic bile duct), age at reconstruction, circumferential or non‐circumferential reconstruction, type of substitute, need for biliary drainage to support the substitute, need for RYHJ, follow‐up, and complications. For experimental studies, the following data were collected: number and type of animals, type of substitute, need for biliary drainage to support the substitute, follow‐up, and survival.

### 2.4 IDEAL Framework and Definitions

Included studies were assigned a stage (0, 1, 2a, 2b, 3, or 4) using the IDEAL guidelines, which describe the stages of surgical innovation: Idea, Development, Exploration, Assessment, and Long‐term follow‐up [16]. “Idea” is the initial stage where a new idea or technique is conceived and applied for the first time. “Development” is the phase during which the procedure is refined through several consecutive cases. “Exploration” is the period during which the technique is disseminated with larger multicenter samples to identify factors of failure and success or to conduct nonrandomized comparative studies. “Assessment” is a phase in which the innovation reaches sufficient maturity for rigorous evaluation through randomized and experimental trials.

“Long‐term follow up” allows the long‐term effects to be studied after widespread adoption of the new technique.

Bile duct replacement using vein grafts and other substitutes is defined as intentional when planned or anticipated preoperatively. Circumferential biliary replacement is defined as a reconstruction in which both ends of the remaining native common bile duct are anastomosed to the substitute (end‐to‐end biliary anastomosis with interposition of a venous graft). Biliary replacements using substitute patches are defined as noncircumferential reconstructions.

## 3 Results

**FIGURE 1:**
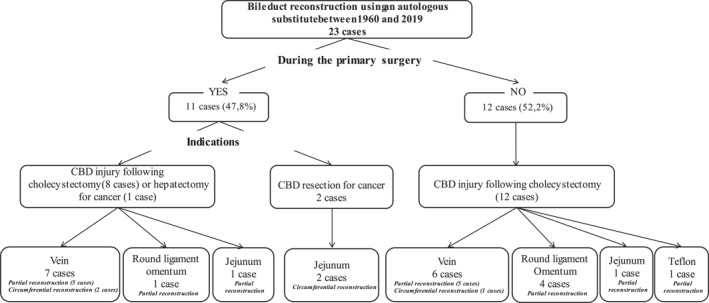
Flowchart of the intervention performed for each of the 23 patients with bile duct replacement using autologous substitutes.

A total of 1135 articles were initially identified: 44 remained after removing duplicates and those that met exclusion criteria (Figure S1). This review included 15 published papers (9 preclinical and 6 clinical studies) in the setting of biliary reconstruction using vein grafts. All the preclinical studies were animal studies, and the majority used mammalian models (Table 1). All the clinical studies were human case reports (Table 2, Figure 1).

**TABLE 1:** Preclinical studies on common bile duct reconstruction using vein grafts.

Author, year, country	Animal/*N*	Substitute	Type of CBD reconstruction	Biliary drain	Postoperative deaths	Survival (followed up until death or sacrifice)	Comments
(Shea 1948) [19], USA	Dog/21	Autologous femoral vein	Circumferential	Stent (vitallium tube)	Early mortality (33.3%): Technical failure (19%) and unknown (14.3)	14 animals (10–208d) with patent grafts including 5 with the presence of biliary epithelium observed on grafts	66.7% of animals had patent grafts
(Pearce 1951) [20], USA	Dog/32	Autologous external jugular vein	Circumferential + Omental wrap (*n* = 25)	Stent + cuffs (vitallium)	Early mortality (93.3%): Technical errors (25%), vein necrosis (34.4%), jaundice (15.6%), perforated duodenal ulcer (12.5%), hepatic abscesses (3.1%), and biliary cirrhosis (3.1%)	2 animals (212–230d) with partial graft stricture without any biliary epithelium observed on grafts	The use of vein grafts to reconstruct the biliary tree in dogs would fail due to necrosis and perforation of the vein, obstruction due to stricture formation, or the precipitation of bile in the vitallium cuffs
(Ulin 1955) [21], USA	Dog/10	Autologous external jugular vein	Circumferential + omental wrap	Stent (polyethylene tube)	Early mortality (20%): Shock and bowel obstruction	8 animals (1–10 mo), including 5 and 1 with partial and slight stricture, respectively, and without any biliary epithelium observed on grafts	Intact viable grafts were observed in all animals. The problem of delayed stricture has been encountered
(Myers 1960) [22],USA	Dog/28	Autologous femoral vein/artery/CBD*	Circumferential	Stent (polyethylene tube)	Marked to complete obstruction of the common bile duct in all cases due to scar tissue	8 animals (< 13d) and 20 animals (51–187d) with obstruction of the graft without any biliary epithelium observed on grafts	Failure of grafts of vein, artery, and common bile duct to replace CBD
(Dunphy 1962) [23], USA	Goat/44 Dog/8	Autologous femoral vein/artery (*n* = 20) homologous artery (*n* = 32)	Circumferential	T‐tube	Early mortality due to technical failure (19.2%)	10 animals (5d–9 mo) with slight stricture but patent grafts without any biliary epithelium observed on grafts	Elevation of serum alkaline phosphatase without stenosis of the graft
(Belzer 1965) [24], USA	Goat/20	Autologous femoral vein	Anterior wall	T‐tube	Early mortality (30%)	14 animals (6d–11 mo) with patent grafts and the presence of biliary epithelium seen on grafts	Viable structure for CBD replacement. At 6 w, the vein patch was incorporated into the wall of the common duct with biliary epithelium. At 5 mo, there is no macroscopic sign of the vein patch
(Lindenauer 1966) [25], USA	Dog/13	Autologous external jugular vein**	Circumferential + Omental wrap	None	Early mortality: Anesthetic overdosage (*n* = 1), necrosis of the graft after stage 1 of 3w (*n* = 2), and small anastomosis at 4w (*n* = 1)	9 animals (mean survival: 3.5 mo) after stage 1 of 8–32w, including 7, which developed progressive jaundice due to the grafts which were noted to be shrunken fibrotic	The 2‐stage CBD replacement produces a viable structure for a short time. Implantation of the vein grafts in the omentum > 8 w avoided early failure due to necrosis of the graft
(Capitanich 2005) [26], Argentina	Rat 12	Autologous femoral vein*	Circumferential	Stent (synthetic)	None	No survival difference between the 2 groups All animals survived at 4 mo with patent grafts and the presence of biliary epithelium seen on grafts	Patent graft and signs of biliary epithelialization at 4 months
(Heistermann 2006) [27], Germany	Pig 12	Autologous external jugular vein	Circumferential	Stent (biodegradable)	None	All animals survived until sacrifice at 6 mo with patent grafts and the presence of biliary epithelium seen on grafts	At 4 mo, the vein grafts had been relined with bile duct epithelium
(Palmes 2009) [28], Germany	Pig 18	Autologous external jugular vein*	Circumferential	Stent (biodegradable)	Stent group: No complications No stent group: Necrosis (*n* = 4), stenosis (*n* = 1), and secondary cirrhosis (*n* = 2)	Stent group: 100% survival at 6 mo No stent group: 33% survival at 6 mo	Patent grafts, stents completely absorbed, and signs of biliary epithelialization seen on grafts at 6 mo

Abbreviations: CBD, common bile duct; d, days; mo, months.

^*^ Comparative study including a control group.

^**^ 2 stage procedure: 1st stage: vein graft harvesting and indirect the vascularization method by inserting the vein graft in the omentum and 2nd stage: CBD replacement using the vascularized vein graft.

**TABLE 2:** Human bile duct reconstructions using different substitutes.

Author, country, year, reference	*N*/age (y)	Indication of bile duct replacement	Substitute	Type of reconstruction	Biliary drainage	Biliary drain removal	Complications	Survival
Venous graft								
Michie [18], UK, 1964	1/36	CBD stricture 36 y after cholecystectomy	Autologous saphenous vein	Partial	T‐tube	NA	None	Alive, 10 mo
Ellis [29], UK, 1980	2/42–58	CDB stricture 1–8 y after cholecystectomy	Autologous saphenous vein	Partial (2/2)	Transhepatic stenting tube (2/2)	2–4 mo	In one patient: Reoperated for extraction of 2 stones in the retropancreatic CBD 4 y later	Alive, 1–7 y
Salim [30], Iraq, 1992	4/42–57	CBD injury during cholecystectomy	Autologous saphenous vein	Circumferential (1/4) partial (3/4)	T‐tube (1/4)	14 d	None	Alive (4/4), 5 y
Watanabe [31], Japan, 2007	4/34–74	CBD injury during cholecystectomy (1/4) and RHD during H4 for cancer (1/4) RHD and CBD 3–7 d after cholecystectomy (2/4)	Autologous umbilical vein	Partial (4/4) reconstruction of the CBD (2/4) and RHD (2/4)	T‐tube + transhepatic stenting tube (3/4) Transhepatic stenting tube + C‐tube (1/4)	T‐tube (30–63 d) C‐tube (31 d) transhepatic tube (0–92 d)	None	Alive (3/4), 44–93 mo Dead (1/4) of tumor recurrence after hepatectomy, 5 mo
Biglari [32], Belgium, 2013	1/61	Bile leakage 6 d after cholecystectomy	Autologous saphenous vein	Circumferential	Preoperative percutaneous drainage left in place postoperatively	5 w	Biliary stenosis treated percutaneously POD48 and prosthesis 2 mo. Stent removal 9 mo	Alive, 12 mo
Rifatbegovic [33], Bosnia, and Herzegovina, 2016	1/51	CBD injury during cholecystectomy	Autologous saphenous vein	Circumferential	None	None	POD3: ERCP with stenting POD13: Biliary leakage. Decision of hepaticojejunostomy	Alive
Jejunum graft								
Wallensten 1960, Sweden [34]	2/64–67	CBD resection for perihilar cholangiocarcinoma type I	Jejunum	Circumferential (2/2)	External drain	NA	N°1: Fever, bile leakage N°2: Hematoma requiring laparotomy, bile leakage	Alive (3–5 mo)
Bengtsson [35], Sweden, 1986	2/49–77	CBD stricture after and CBD injury during cholecystectomy	Jejunum	Partial (2/2)	T‐tube (2/2)	6 w	None	Alive, 1 and 7 y
Prosthetic graft								
Thomas, USA (37), 1963	1/32	Recurrence after by bilio‐biliary anastomosis for CBD stricture after cholecystectomy	Teflon	Partial	T‐tube	2 y	None	Alive, 48 mo
Round ligament and omentum grafts								
Chang [36], USA, 2000	1/32	BDI during cholecystectomy	Round ligament	Partial	T‐tube	3 mo	None	Alive, 12 mo
Dokmak [37], France, 2017	2/33–59	BDI after cholecystectomy	Round ligament	Partial (2/2)	T‐tube (2/2)	3–6 mo	None	Alive, 6–9 mo
Ng [38], Singapore, 2017	1/29	BDI 24 d after cholecystectomy	Omentum	Partial	None	—	Hepaticojejunostomy 3 mo later	Alive
Irigoyen [39], Brazil, 2019	1/58	BDI after cholecystectomy	Round ligament	Partial	T‐tube	6 weeks	None	Alive

Abbreviations: BDI, bile duct injury; CBD, common bile duct; d, days; H4, segmentectomy 4; mo, months; NA, not available; POD, postoperative day; RHD, right hepatic duct; w, weeks; y, years.

### 3.1 Study Characteristics and IDEAL Stage

None of the six clinical studies reported an IDEAL stage. All studies were classified as IDEAL stage 1. The first publication of early experiences with biliary reconstruction using an isolated jejunal segment was in 1960 and originated from Sweden [34]. The first publication of biliary reconstruction using a venous graft was in 1964 and originated from the United Kingdom [18].

### 3.2 Stage 0: Pre‐IDEAL

The pre‐IDEAL stage is the preclinical phase in which the feasibility of a new surgical technique is first evaluated in preclinical animal studies. These preclinical studies mainly used large mammals and biliary substitutes derived from autologous tissues, synthetic prostheses, and tissue engineering. The first experimental model of biliary reconstruction using a biliary substitute was reported in 1900, when Sulivan et al. inserted a rubber tube between the main bile duct and duodenum in dogs [1]. The first experimental model of biliary reconstruction using a vein graft was performed in dogs in 1918 by Horsley et al. [40]. Other preclinical animal studies showed that autologous arterial grafts [41], gallbladder flaps [42], serosa [43], skin [44], and ureters [45] were suitable biliary substitutes (Supporting Information S1: Tables 1–3). Table 3 summarizes the different graft types and their advantages and limitations.

**TABLE 3:** Summary of substitutes for bile duct replacement.

Substitutes	Description	Advantages	Inconvenient	Limitations
Autologous venous grafts	Patient's own tissues	No risk of rejection Biocompatible Long‐term stability	Lack of « biliary clearance »	Long‐term stenosis
Allografts	Decellularized tissues from cadaveric donors	Availability No risk of rejection Biocompatible Long‐term stability	Lack of “biliary clearance” Availability	Long‐term stenosis
Synthetic prosthesis	Nonbiocompatible materials (silicone, dacron…)	Availability	Risk of infection Not biocompatible Potential for bile leakage	Poor long‐term viability High risk of complications (stenosis, infection)
Bioabsorbable tissue engineering	Creation of bile duct tissues using a bioabsorbable scaffold	Biodegradable and absorbable tissue	Availability Still experimental	Insufficient evidence of tissue regeneration High experimental failure rate

#### 3.2.1 Characteristics of the Included Studies

The preclinical animal studies used dogs (*n* = 91; 46.2%), goats (*n* = 64, 32.5%), pigs (*n* = 30, 15.2%), and rats (*n* = 12, 6.1%), including test and control animals in comparative studies. Most studies reported outcomes for a group of animals using the first animals to optimize the surgical technique. In eight of the nine studies, the common bile duct was resected followed by circumferential common bile duct reconstruction using an autologous vein graft, either the external jugular (*n* = 79, 53.4%) or femoral (*n* = 69, 46.6%) vein [19, 20, 21, 22, 23, 24, 25, 26, 27, 28]. One study performed noncircumferential common bile duct reconstruction using a vein patch [24]. Another study reported two‐stage common bile duct reconstruction: in stage 1, the vein graft was inserted in the omentum, and in stage 2, the common bile duct was reconstructed [25]. Wrapping techniques using omentum for indirect vascularization of vein grafts were used in two studies [21, 25]. The vein graft was supported by an internal drain or stent in seven studies [19, 20, 21, 22, 26, 27, 28] and by an external T‐drain in two studies [23, 24].

#### 3.2.2 Survival After Common Bile Duct Reconstruction Using an Autologous Vein Graft

The development of preclinical animal studies can be divided into three periods (Table 1). In 1948, Shea et al. were the first to use a stent (vitallium tube) to support an autologous femoral vein graft for circumferential common bile duct reconstruction in 21 dogs to prevent biliary stenosis [19]. They reported a postoperative mortality rate of 33.3% mainly due to technical failure. However, *de novo* generation of biliary epithelium was observed within the vein graft in 5 of the 14 surviving animals. Pearce et al. confirmed these results in 1951, observing a mortality rate of 93.3% in dogs [20]; in 1960, Myers et al. observed complete obstruction of the reconstruction in all the 28 cases [22]. It is interesting to note that vein grafts were actually used in the two studies that encountered delayed biliary strictures [21, 23].

#### 3.2.3 De Novo Biliary Epithelium Regeneration

Histological analysis provides valuable data that can reveal potential biliary epithelialization of the substitute and therefore evidence of its long‐term viability. However, these data can only be available in animal sacrificed for analysis and not in human cases. Tissues identified as neoepithelium were isolated and used for histological studies.

In their preclinical model of partial reconstruction of the anterior wall of the common bile duct using an external jugular vein in 1965, Belzer et al. were among the first to describe the *de novo* generation of biliary epithelium within the vein graft from 6 weeks to 5 months [24].

In the 2000s, three animal studies using pig and rat models showed that autologous vein grafts could be substitutes for common bile duct reconstruction. The de novo generation of biliary epithelium was observed within the vein graft at 4 months [26, 27, 28].

In the evaluable animals, histological examination revealed an internal surface focally lined with biliary epithelium in four studies [19, 26, 27, 28].

### 3.3 Stage 1: Idea

Stage 1 includes the first‐in‐human reports (Figure 1). In this stage, the new surgical procedure was performed on a few highly selected patients, with the outcomes detailed in case reports. Although the first circumferential biliary reconstruction to be reported as a first‐in‐human study was biliary reconstruction using an isolated jejunal segment following common bile duct resection for cancer in 1960, safety concerns and technical complexity led surgeons to abandon further work on this technique [34, 35]. The use of jejunal substitutes was eventually abandoned, probably for technical and infectious reasons. In 1963, Thomas et al. performed the first‐in‐human biliary noncircumferential replacement using a substitute patch of Teflon for a stricture that developed 7 months after a biliary repair of a postcholecystectomy bile duct injury. This patient has remained asymptomatic 2 years later. To date, only one human case involving synthetic material has been reported, probably for infectious reasons [46]. In 1962, Michie et al. performed the first‐in‐human biliary reconstruction using a vein graft, with partial reconstruction of the common bile duct using a vein graft for a stricture that developed 26 years after a cholecystectomy [18]. In 1992, Salim et al. performed the first‐in‐human circumferential biliary reconstruction using a venous graft for a common bile duct injury sustained during cholecystectomy [30].

To date, 23 patients have undergone biliary reconstruction using either autologous substitutes such as venous grafts (*n* = 13, 56.5%), round ligament/omentum (*n* = 5, 21.7%), and jejunum (*n* = 4, 17.4%) or prosthetic grafts (*n* = 1, 4.3%). Table 2 [18, 29, 30, 31, 32, 33, 34, 35, 36, 37, 38, 39, 46] summarizes the 23 cases (reported in the period 1964–2019) of bile duct reconstruction using autologous grafts (round ligament, venous graft, omental patch, or gallbladder), including the 13 cases that used a vein graft as an autologous substitute between 1960 and 2016. The majority of procedures were performed for benign indications (to repair a stricture due to an injury following cholecystectomy). Only two cases involved cancers. Biliary reconstruction using a bile duct substitute was considered intentional in 10 (43.5%) cases.

#### 3.3.1 Feasibility of Biliary Reconstruction Using a Vein Graft

All attempts at biliary reconstruction using a vein graft were successful in all patients (mean age 51 years). The indications were bile duct injury or stricture following cholecystectomy (*n* = 12, 92.3%) and liver resection for perihilar cholangiocarcinoma (*n* = 1, 7.8%). Biliary reconstruction using a vein graft was performed intraoperatively (*n* = 7, 53.8%) or postoperatively (*n* = 6, 46.2%). For those performed postoperatively, reconstruction was performed within 14 days in three cases [31, 32] and after 6 weeks in three cases [18, 29].

All biliary reconstructions were performed using autologous venous grafts, including saphenous (*n* = 9, 69.2%) and umbilical (*n* = 4, 30.8%) veins. The surgical options for biliary reconstruction range from lateral patch repair (*n* = 8, 61.5%) to full‐circumferential common bile duct reconstruction (*n* = 5, 38.5%). Vein grafts were supported by a biliary drain in 92% of the cases. The delay between the biliary reconstruction and biliary drain removal ranged from 14 to 120 days. Twelve patients were alive at the last follow‐up (Table 2). No histological studies of biopsies or brushing were performed to observe regeneration or de novo generation of the biliary epithelium.

#### 3.3.2 Safety

The mean follow‐up was 41 months, and there was no 90‐day mortality, although two patients (15.4%) had 90‐day morbidity: one case of biliary stenosis and one of biliary leakage requiring RYHJ on postoperative day 13 [33]. The latter patient underwent circumferential common bile duct reconstruction using an autologous saphenous vein without any external biliary drainage for a bile duct injury sustained during cholecystectomy. This patient was alive at the last follow‐up. In the intermediate term, one patient had a stone extracted surgically 4 years later [29] and one patient with biliary stenosis was treated percutaneously by biliary drainage on postoperative day 48 and by a stent on day 60 [32]. No late cholangitis due to reflux was reported during the study period (Table 2). Twelve (92.3%) patients were alive at the last follow‐up. One patient who had been operated on for a cancer died of liver recurrence 5 months after liver resection [31]. The follow‐up periods for the various studies vary considerably (from 3 months to 5 years) (Table 2).

### 3.4 Stage 2A: Development

The evolution of this technique has yet to reach stage 2A according to the IDEAL framework. At this stage, common bile duct reconstruction using venous grafts should be performed in an initial small (usually < 30) group of prospectively recruited patients in a single institution, with the short‐term clinical, technical, safety, and reproducibility outcomes being reported. So far, there have been no reports beyond stage 1. The challenges in designing stage 2 studies include technical considerations (should the venous graft be supported by a biliary drain?), appropriate indications (malignant or benign hilar stenosis, nonhilar malignant or benign strictures, liver transplantation, and choledochal cyst), and the primary outcomes studied (absence of reintervention with hepaticojejunostomy within 90 days). We found no ongoing clinical trials in searches of clinical trial databases.

## 4 Discussion

### 4.1 Indications for Such a Procedure

RYHJ is the standard for reconstruction of the biliary tract. However, this nonphysiological reconstruction of the biliary tract has some drawbacks in the short‐term (ascending cholangitis and biliary strictures) and the long‐term (secondary biliary cirrhosis due to chronic inflammation and repeated cholangitis). More importantly, RYHJ limits access to the biliary tree for endoscopic intervention in cases with biliary complications.

From a theoretical standpoint, common bile duct replacement, which is a physiological reconstruction of the biliary tract, may have valuable advantages: (1) the added benefits of postoperative endoscopic access to the biliary tree, particularly for the treatment of biliary complications and (2) avoiding ascending cholangitis and its long‐term effects because the presence of the sphincter of Oddi prevents reflux of intestinal contents and bacteria in the biliary tract.

### 4.2 Experience With Human Bile Duct Replacement Using Venous Grafts and Other Substitutes

This paper provides an overview of all studies in which bile duct replacement using various substitutes was attempted in humans. Twenty‐three human cases were collected and were heterogenous in terms of the indications and biliary substitutes used. In the majority of cases, the technique was used to treat bile duct injury following cholecystectomy. More than half of the cases were performed between 1960 and 1999.

The technique shifted from using various biliary substitutes to autologous substitutes due to the many advantages of the latter (Table 3). In the 2000s, bile duct replacement used only autologous substitutes either venous grafts or peritoneum/round ligament. The outcomes of this innovative surgical procedure are somewhat acceptable. More importantly, the 90‐day mortality rate was zero. The 90‐day bile leakage rate and the proportion of patients who required conversion to RYHJ were 4.5% and 9%, respectively.

Venous grafts were the most frequently used substitutes (56.5%) for bile duct reconstruction. Focusing specifically on venous grafts as autologous substitutes for bile duct reconstruction, the rate of 90‐day bile leakage and the proportion of patients who required conversion to RYHJ were < 10%, which was acceptable compared to the bile leakage rates following liver resection for perihilar cholangiocarcinoma [47] and surgical repair of bile duct injury following laparoscopic cholecystectomy (early repair: 10.5% and delayed repair: 4.8%) [48]. The advantages of a venous graft over peritoneum, round ligament, or jejunum include the fact that this is a tubular substitute with intrinsic lateral and longitudinal rigidity and flexibility and an adequate lumen, and there is no need to recreate a functional tube from the patient's own tissues. Regarding the efficacy and long‐term results of bile duct reconstruction using various substitutes, several reports lack clear documentation of the postoperative follow‐up. Of note, no human data on the epithelial coverage was provided. Another important point in bile duct reconstruction concerns the differences between circumferential and noncircumferential applications. Here again, venous grafts may be the simplest option: they do not require free flap technique, and all circumferential human bile duct replacements (4/4) used autologous venous grafts rather than peritoneum/round ligament (0/4). This may be important for two reasons: in noncircumferential reconstruction, the graft can be vascularized contiguously by the remaining native common bile duct membrane, whose distal vascularization has not been damaged; and reepithelialization can be achieved from the native common bile duct. By contrast, in circumferential reconstruction, in the absence of a surgical strategy (such as the wrap technique), only the edges of the graft can provide vascularization and epithelial cell migration.

However, this systematic review is composed of case reports and only 23 cases of bile duct replacement have been reported over 6 decades, which reflects limited clinical need and technical challenges. More importantly, the widespread adoption of this technique of bile duct reconstruction has been limited because RYHJ is the standard for reconstruction of the biliary tract.

### 4.3 Experience With Animal Bile Duct Reconstruction Using Venous Grafts and Other Substitutes

The literature on bile duct substitutes is very heterogeneous, with the vast majority of animal studies involving different species, surgical techniques, and types of substitutes. This makes it very difficult to come to a conclusion about the best substitute to use, but there are several potential clues. The use of autologous venous grafts initially produced mixed results, with a high rate of biliary fistula and graft stenosis. The early experimental trials in dogs conducted in the 1950s and 1960s are difficult to interpret due to methodological differences [19, 21, 22, 23]. More recent trials in the 20th century, particularly in pigs, produced much more satisfactory results, which can be explained by the use of an intraluminal stent or T‐drain. German teams [27, 28] proposed the use of a biodegradable stent inside the venous graft to avoid the complications associated with metal or plastic stents, including occlusion, migration, and kinking.

Vascular prostheses had poor results, with biliary fistulas and stenosis being linked to an intense inflammatory reaction. The nonbioabsorbable nature of these substitutes triggers an inflammatory reaction with a fibrotic reaction around the prosthesis. These prostheses often require anticoagulation to prevent clot formation. Bile is even more viscous than blood and has a lower flow rate [49], so it potentially confers an even greater long‐term risk of clot or stone formation, especially if an intense inflammatory reaction causes stenosis. Therefore, bile duct substitutes made from nonbioabsorbable synthetic materials do not appear to be suitable for reconstructing the common bile duct in clinical practice.

Bioabsorbable substitutes, either bioengineered synthetic tissues or decellularized allografts, are being increasingly discussed in the literature. Despite the heterogeneous materials and methods used, the preliminary results are satisfactory, with few surgical complications in animals and absorption and, in some cases, biliary reepithelialization of the graft. Recently, Montalvo‐Jave et al. developed the most comprehensive protocol involving multidisciplinary assessment, testing an absorbable collagen‐based polymer in two exploratory cohorts of pigs with satisfactory long‐term results [50]. Li et al. proposed using 3D printers to develop bioabsorbable polymer tubes cultured with stem cells as potential bile duct substitutes [51]. In 2020, Sampaziotis et al. reported the use of organoid cholangiocytes to regenerate the intrahepatic bile ducts of deceased donor liver grafts on a normothermic machine [52]. They observed proliferation of these organoids in the bile ducts. However, the use and development of these substitutes is extremely complex and is at a very early stage of development. Their use in human clinical practice in the near future seems uncertain.

### 4.4 Biliary Drainage to Support Biliary Reconstruction

**FIGURE 2:**
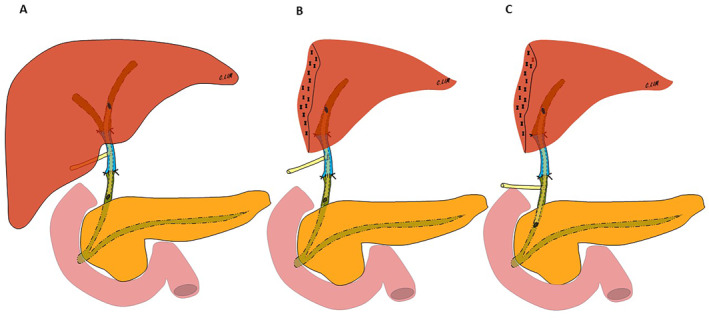
Schematic illustrations of biliary drainage using a T‐tube after bile duct replacement using an autologous vein graft in hepatobiliary surgery. (A) After resection of the common bile duct, the latter was replaced by a vein graft with a T‐tube inserted through the vein graft via a venotomy. (B) After right‐sided hepatectomy and common bile duct resection, the latter was replaced by a vein graft with a T‐tube inserted through the vein graft via a venotomy. (C) After right‐sided hepatectomy and common bile duct resection, the latter was replaced by a vein graft with a T‐tube inserted through a choledochotomy.

Venous grafts and omentum/round ligament have one major conceptual difference: the use of biliary drainage. It is easier to place a T‐drain or internal biliary stent within the lumen of a venous graft than a flap of peritoneum/round ligament where a stent or T‐drain might narrow the lumen and cause biliary obstruction. Another important point is that the stent would be used to reinforce the architecture. The theoretical advantages of T‐drain placement include passive decompression and drainage of the biliary tract and postoperative access to the vein graft for cholangiography. However, the question remains: how long do we have to keep the stent or T‐drain (Figure 2)?

### 4.5 What Will Be the Best Substitute?

The ideal biliary substitute should be able to integrate the surrounding host tissues without complications (inflammation, granulation tissue formation, and infection). It should be rapidly available, easily to be harvested, biocompatible, nontoxic, nonimmunologic, noncarcinogenic, and durable (avoiding stenosis or erosion, Table 3). To determine the best features of biliary substitutes, it is of utmost importance to understand the mechanisms of biliary repair, including the dynamics of cell colonization and reepithelialization. Given that current applications are scarce, preclinical studies with a relevant animal model similar in size and anatomy to humans with sufficiently long follow‐up are required to draw robust conclusions on the biointegration of and changes in biliary substitutes over time. Porcine models seem to be the reference model to facilitate obtaining approval from regulatory authorities and should be used as such: their size is adequate and their immune system is known.

To gain a clearer picture of these mechanisms, it might be useful to standardize follow‐up criteria and include more detailed biological and molecular evaluations to allow the correlation of biological markers with final outcomes: biomechanical integrity over time, a histological analysis that scores vascularization and reepithelialization with biliary cells, the detection of granulation tissue and stenosis, immune cell infiltration, clinical outcomes with endoscopic treatments, and the need for stenting. No bioengineered biliary allografts have been developed for humans. Although appealing, this fascinating technique is still far from clinical application, involving seeding of the patient's own stem cells in a 3D biliary matrix, which is then incubated in a bioreactor preoperatively.

In conclusion, compared to the clinical experience with stented aortic allografts for tracheal replacement [53, 54], bile duct reconstruction using vein grafts remains experimental (IDEAL stage 2a). Several secondary aspects of this procedure are yet to be defined, that is, restoring fully functional biliary drainage with biliary epithelium, without the need for long‐term stenting. Besides the inherent risks associated with an innovative surgical procedure, the need for long‐term biliary stenting remains unclear.

## Author Contributions

**Mehdi Boubaddi:** conceptualization, data curation, investigation, methodology, writing – original draft, writing – review and editing. **Chetana Lim:** conceptualization, data curation, investigation, methodology, project administration, software, supervision, validation, visualization, writing – original draft, writing – review and editing. **Eric Savier:** project administration, validation, writing – review and editing. **Claire Goumard:** project administration, validation, writing – review and editing. **Florence Jeune:** project administration, validation, writing – review and editing. **Geraldine Rousseau:** validation, writing – review and editing. **Filomena Conti:** writing – review and editing. **Fabiano Perdigao:** project administration, writing – review and editing. **Olivier Scatton:** conceptualization, project administration, supervision, validation, visualization, writing – review and editing.

## Conflicts of Interest

The authors declare no conflicts of interest.

## Supporting information


Supporting Information S1

**Figure S1**: Flowchart of the systematic review.

## Data Availability

The authors have nothing to report.
