# Supplementary material for: Bile Duct Replacement in Hepatobiliary Surgery: A Systematic Review
Source: World J Surg. 2025 Sep 24;49(11):3182–94. doi: 10.1002/wjs.70078 (PMC12582139; doi:10.1002/wjs.70078)
Supplement: Supplementary file 1 — Supporting Information S1 [file WJS-49-3182-s002.docx]

| **Supplementary Table 1. Preclinical studies on common bile duct reconstruction using synthetic grafts** | | | | | | | |
| --- | --- | --- | --- | --- | --- | --- | --- |
| **Author, year, country** | **Animal/ N** | **Substitute** | **Type of CBD reconstruction** | **Biliary drain** | **Postoperative deaths** | **Survival (followed up until death or sacrifice)** | **Comments** |
| Bergan et al, 1962, USA | Dogs/  21 | Vascularized polyvinyl sponge | Circumferential | None | Early mortality = 80.9% | 4 dogs survived after 60 days (maximum 14 months) with stenosis of anastomosis | - Intense inflammatory reaction around the graft  - Excessive early mortality |
| Sherman et al, 1963, USA | Dogs/  37 | Acrylate Amide Vascular Prosthesis | Circumferential | None | Early mortality = 48.6%  4 deaths from biliary leakage and 1 cholangitis | - 19 dogs alive after a month  - 4 dogs alive after 19 months (maximum 31 months) | - Intense inflammatory reaction around the graft without biliary epithelialization |
| Gulati et al, 1982 | Dogs/  20 | Dacron Velour | Lateral patch | None | No postoperative death  Regular sacrifice until day 485 | - Liver enzymes normalization  - Regular sacrifice until day 485 | - Intense inflammatory reaction around the patch |
| Mendelowitz et al, 1983 | Dogs/  11 | Gore-Tex vascular graft or Dacron | Circumferential | None | 3 postoperative deaths from surgical complication  Sacrifice at 30-40 days | -8 dogs survived before sacrifice at day 30-40 | - Intense inflammatory response  - Kinked, bile stained, partly or completely rejected  - No biliary epithelialization |
| Gomez et al 2002, Ecuador | Dogs/  12 | Gore-Tex vascular graft | Circumferential  Hepaticoduodenal | None | No postoperative death  Regular sacrifice at 1, 2 and 3 months | 100% survival at M1, M2, M3  Normalization of liver enzyme  No stenosis or leakage | - No long-term results  - No biliary epithelialization  - Intense inflammatory response |
| Christensen et al, 2005, Denmark | Pigs/ 8 | Vascular prosthesis expensive polytetrafluoroethylene (ePFTE) Teflon 4mm | Circumferential, prothesis invaginated in main bile duct | None | Sacrifice at day 8 | 100% survival at sacrifice  No liver biology impairment  1 case of biliary leakage at sacrifice | - No long-term results  - No biliary epithelialization  - Intense inflammatory response |
| Napolitano et al, 2015 Argentina | Pigs/ 9 | Temporary Silicone Tube before RYHJ | Circumferential | None | No postoperative death | 100% survival at laparotomy (60 days)  No liver biology impairment  No cholangiography impairment | Feasibility of second procedure for biliodigestive anastomosis |
| **Footnotes:**  CBD indicates common bile duct. | | | | | | | |

| **Supplementary Table 2. Preclinical studies on common bile duct reconstruction using bioabsorbable grafts** | | | | | | | |
| --- | --- | --- | --- | --- | --- | --- | --- |
| **Author, year, country** | **Animal/ N** | **Substitute** | **Type of CBD reconstruction** | **Biliary drain** | **Postoperative deaths** | **Survival (followed up until death or sacrifice)** | **Comments** |
| Miyazawa et al, 2005, Japan | Pigs/ 18 | Bioabsorbable polymer tube | Circumferential  Hepaticoduodenal | None | No postoperative death  Regular sacrifice at 6, 10, 24 months | 100% survival at sacrifice  No stenosis or leakage  No liver biology impairment | Biliary epithelialization at 6 weeks |
| Ismail et al, 2009, Egypt | Dogs / 40 | Human amniotic membranes on peritoneomuscular flap | Lateral patch  Amniotic membrane alone (A n = 20) vs combined with peritoneomuscular flap (B n=20) | None | - 3 early postoperative death for leakage in group A  - No surgical complication in group B  - Sacrifice at 6 weeks | - 100% survival at sacrifice in group B  - 85% survival at sacrifice in group A | Biliary epithelialization at 6 weeks on amniotic membrane |
| Aikawa et al, 2011, Japan | Pigs / 10 | Bioabsorbable synthetic polymer tube | Circumferential | Silicone stent | - No postoperative early complication  - Sacrifice at 4 months | - 100% survival at sacrifice  - No liver biology impairment  - No stenosis or leakage | - Biliary epithelialization at 4 months |
| Nau et al, 2011, USA | Dogs/ 11 | Bioabsorbable synthetic polymer  (polyglycolic acid and trimethylene carbonate) | Circumferential + omentoplasty | Plastic stent | - Early mortality = 54.5% (n = 6)  1 leakage and 5 cholangitis | - 45.5% survival at sacrifice (6, 7, 8, 12 months)  - 2 successful endoscopic stent removal at 6-8 weeks | Biliary epithelialization at 6 months |
| Liang et al, 2012, China | Pigs / 8 | Bioabsorbable synthetic stent of polymer | Circumferential + omentoplasty | None | No postoperative death  Sacrifice at 3 and 12 months | 100% survival at sacrifice  No leakage or stenosis | Complete absorption and biliary epithelialization at M4 |
| Tao et al, 2015, China | Pigs/ 20 | Collagen patch scaffold | Lateral patch + omentoplasty | None | No postoperative death  Sacrifice at 2, 4, 8 and 12 weeks | 100% survival at sacrifice  No leakage or stenosis | Complete absorption at 8 weeks |
| Tanimoto et al, 2016 | Pigs/ 11 | Radiopaque absorbable biliary stent  Copolymer L-lactide and caprolactone and coated with barium sulfate | Circumferential | None | No postoperative death  Sacrifice at 6 months | 100% survival at sacrifice  No leakage or stenosis  Normal liver biology and CT scan at 6 months | Complete absorption and epithelialization at M6 |
| Zhong et al, 2017, China | Pigs / 18 | Polycaprolactone /Polylactide-co-glycolide polymer with human mesenchymal stem cells (HMSC) | Circumferential | None | No postoperative death  Sacrifice at 4, 12 and 24 months | 100% survival at sacrifice  No leakage or stenosis | Better biliary epithelialization and repair in HMSC group at 2 months |
| Montalvo-Jave et al, 2015, Mexico | Pigs/ 15 | Absorbable bio prosthesis polymer based (collagen) | Circumferential | None | No postoperative death  Sacrifice at 6 months | 100% survival at sacrifice  No leakage or stenosis at ECRP, spyglass and bili MRI at 6 months | Prosthesis biliary epithelialization at 6 months  No complete absorption |
| de Abreu et al, 2020, Brazil | Pigs/ 20 | Bacterial cellulose film | Lateral patch | None | No postoperative death  Sacrifice at day 150 and 330 | 100% survival at sacrifice  No leakage or stenosis | No biliary epithelialization |
| Montalvo- Jave 2024, Mexico | Pigs/ 16 | Bio prosthesis absorbable polymer based (collagen) | Circumferential | None | No postoperative death  Sacrifice at 24 months | 100% survival at sacrifice  No leakage or stenosis at ECRP, spyglass and bili MRI at 24 months | Complete absorption and epithelialization at 24 months |

| **Supplementary Table 3. Preclinical studies on common bile duct reconstruction using allografts** | | | | | | | |
| --- | --- | --- | --- | --- | --- | --- | --- |
| **Author, year, country** | **Animal/ N** | **Substitute** | **Type of CBD reconstruction** | **Biliary drain** | **Postoperative deaths** | **Survival (followed up until death or sacrifice)** | **Comments** |
| Rosen et al, 2002, USA | Dogs/ 15 | Decellularized porcine small intestine submucosa (SIS) | Lateral patch | None | Early postoperative death = 6.7% (n=1, biliary leakage)  Monthly sacrifice until 5 months | 93.3% alive at sacrifice (n=14)  No leakage or stenosis | Complete absorption and epithelialization at 24 months |
| Cheng et al, 2016, China | Pigs/ 18 | Decellularized porcine ureters | Circumferential | T drain (n = 6)  Silicone stent (n=6)  None (n=6) | Early mortality: 100% without stent or drain (1 leakage and 5 stenosis)  Sacrifice at 3 months | 100% survival in T drain and stent group (n=12)  No leakage or stenosis  0% survival at 2 months in the group without stent or drain | Prosthesis biliary epithelialization at 3 months |
| Shang et al, 2020, China | Pigs/ 8 | Decellularized bovine ureters | Circumferential | None | No postoperative death  Sacrifice at 6 and 12 months | 100% survival at sacrifice  1 case of biliary leakage at sacrifice  No case of stenosis | Prosthesis biliary epithelialization at 6 months |
