# Supplementary material for: Bile Duct Replacement in Hepatobiliary Surgery: A Systematic Review
Source: World J Surg. 2025 Sep 24;49(11):3182–94. doi: 10.1002/wjs.70078 (PMC12582139; doi:10.1002/wjs.70078)
Supplement: Supplementary file 2 — Figure S1: Flowchart of the systematic review. [file WJS-49-3182-s001.docx]

**Supplementary figure 1:** flow chart of the systematic review
